# Veterinary Science in Europe

**Published:** 1860-05

**Authors:** 


VETERINARY SCIENCE IN EUROPE.—We are glad to perceive that Queen Victoria was induced last year to grant a charter to the veterinary surgeons of Great Britain, constituting them a legally recognized profession. This desirable object was brought about chiefly, it would seem, through the influence and exertion of Mr. John Gamgee, one of the professors in the New Veterinary College of Edinburgh. From the *Edinburgh Veterinary Review and Annals of Comparative Pathology*, a quarterly journal, the January number of which is now before us, we learn that thirty-nine students are in attendance at the new college, and the journal itself affords evidence of being conducted with unusual spirit and ability. We see that Edinburgh has a veterinary instrument-maker, and a firm which puts up veterinary medicine chests, accompanied by a "treatise and table of doses." It has also recently issued the following works on the subject: "The Veterinarian's Vade-Mecum," by Professor John Gamgee;

"Veterinary Medicines; their Actions and Uses," by Finlay Dun; "Stable Economy," by John Stewart; "Advice to Purchasers of Horses." In our Periscopic department will be found a short extract on emboli in the horse, from the January number of the *Edinburgh Review*, which will, we doubt not, interest many of our readers.

We trust that the time is not distant when the people of this country shall become thoroughly aroused upon the subject of veterinary medicine and surgery; a subject of the deepest interest to the agriculturist of the United States, and to all humane and enlightened citizens. Every physician should take a personal interest in the matter, and do all he can with the farmers and stock-growers in his neighborhood to promote the establishment of veterinary schools.

